# 

**Published:** 1891-01

**Authors:** 


### INDEX

TO THE

### HOMŒOPATHIC PHYSICIAN.

### VOLUME XI.

PAGE

Aceraceæ, 168

Action and Reaction, 15

Advice Wanted. A. T. Noe, M. D., 254

Advice to Women. By J. Adams, M. D. Review of, 91

American Institute of Homœopathy, 300

Report of Bureau of Organization, etc., 44

American Institute of Homœopathy and the International Homœopathic Congress, The. Pemberton Dudley, M. D., 121

American Public Health Association, The. Notice of, 376

Anæsthetics in Labor. Bureau of I. H. A., 95

Analysis Sheet, The Hahnemannian's. Alfred Heath, 288

Anatomy, Treatise on. Review of, 476

Aneurism—Cases from Practice. Prof. Edmund Carleton, M. D., 380

Annals of Surgery. Notice of, 92, 475

Announcement of the International Medical Annual, 135

Annual Report of the Postmaster-General of the U. S., year 1890. Notice of, 132

Arnica, Medicinal Shock. Wm. Jefferson Guernsey, M. D., 248

Arnica, 423

Arsenic for Common Use, 285

Arsenic Poisoning, 169

Ars., 423

Baby Food, 335
Bacterial Diseases (So-called), Curative Treatment of. Samuel Swan, M. D., 241
Bakody, Prof., Scientific Medicine, etc., 475
Balm of Gilead Buds, An Accidental Proving of. W. C. Stilson, M. D., 88
Baryta-carb., 220, 421, 430
Bates' Numbering Machine, 478
Belladonna, 214, 428

PAGE

Bell, James B., M. D. An Open Personal Letter to the Members of the I. H. A., 434
Bender, Dr. Note, 344
Berridge, E. W., M. D. Clinical and Pathogenic Notes, 218
Bœnninghausen's Therapeutic Pocket-Book. By Dr. T. F. Allen. Review of, 91
Bœnning, H. C., M. D. Treatise on Practical Anatomy, 476
Boger, C. M., M. D. Gonorrhœa, with Shotgun Treatment, 400
Book Notices, 42, 90, 132, 182, 228, 262, 291, 339, 372, 407, 437, 474
Book for Advertisers. Geo. P. Rowell & Co., 292
Boston Hahnemannian Association, The, Notice of, 183
Bradford, Dr. Thos. L. Homœopathic Bibliography of the U. S., 263
Brady, Ed. F., M. D. The Teaching of Homœopathy in the Colleges, 39
British Medicinal Plants. Alfred Heath, M. D., 19, 79, 164, 200, 251, 368, 468
Burnett, J. Compton, M. D. Greater Diseases of the Liver, 476

Calc-carb., 420, 424

Carb-veg., 420

Carduus Marianus. Dr. H. Kunze. Translated by S. L., 221

Carleton, Prof. Edmund, M. D. Aneurism—Cases from Practice, 380

Case, A Queer. T. Dwight Stow, M. D., 212

Cases from Practice. Dr. Lorbacher. S. L., 224

Cash, Nathan, M. D. Indigestion in Infants, 394

Celastraceæ, 200

Census Bulletins, Notice of, 44, 132, 291

Chamomilla, 220

Cheltenham Reveille, 475

Children's Homœopathic Hospital, 184

PAGE
Chloroform Treatment in Typhoid Fever. Dr. Hepp. S. L., . . . . . . 181
Choate, Dr. Rufus, . . . . . . . . . . . 477
Cholera Infantum. Wm. Steinrauf, M. D, . . . . . . . . . . . . . . . 470
Chronic Intoxication from the Essences, as Wermuth, etc. S. L. 466
Clark, Geo. H., M. D.
The Development of Nosode Practice in the Old School, . . . . . 6
The Doctor, . . . . . . . . . . . . . 345
The Future of Medicine, . . . . . 8
Heart Failure, . . . . . . . . . . . . 267
The I. H. A. Meeting, . . . . . . . 297
Infant Food, . . . . . . . . . . . . 237
Government Indorsement of Professor Koch, . . . . . . . . . . . . 190
"Higher Medical Education," . . 139
Koch's Lymph and Swan's Tuberculinum, . . . . . . . . . . . . . 185
Miscarriage, . . . . . . . . . . . . 265
Note, . . . . . . . . . . . . . . . . 403
Ophthalmia Neonatorum, . . . . . 266
Professor Koch's Discovery, . . . . 5
The Psora Theory, . . . . . . . . . 93
The Psora Theory and Dr. Reuter's Observations, . . . . . . . . . . 237
Salutatory. Editorial, . . . . . . . 4
Superstitions, . . . . . . . . . . . 233
Dr. Swan's Theory, . . . . . . . . 5
Clarke, Dr. Wm B. Homœopathy and Blood Letting, . . . . . . . . . . 43
Cleveland Homœopathic Hospital College, . . . . . . . . . . . 136, 231, 478
Clinical and Pathogenic Notes. E. W. Berridge, M. D., . . . . . . . . . . 218
Clinical Guide. G. H. G. Jahr, . . . . 474
Clinical Verifications. George F. Dunham, M. D., . . . . . . . . . . . . 220
Clinical Verifications. Geo. W. Sherbino. M. D., . . . . . . . . . . . . 69
Cobb, Dr. Harriet H., . . . . . . . . . 477
Cohen, S. W., M. D. Renal Colic, Berberis-vulg., . . . . . . . . . . . . 431
College, Cleveland Medical, . . . . . . 479
College, Long Island, . . . . . . . . . 480
College, National Homœopathic Medical, Chicago, . . . . . . . . . . . 478
College of Physicians and Surgeons, . 477
Commencement of Cleveland Homœopathic Hospital College, Notice of, . . . . . . . . . . . . . . . . . 231
Commencement of the Homœopathic Medical College of Missouri, The, . . . . . . . . . . . . . . . . 231
Compend of Anatomy and Physiology. Review of, . . . . . . . . . . . . . 373
Confinement, The Treatment of Women in. Proceedings of the I. H. A., . . . . . . . . . . . . . . . . . 73
Conglomerate, The, Notice of, . . . . . 439
Consumption. Five Years' Experience in the New Cure of, . . . . . . . . 182
Corrections, . . . . . . . . . . . 92, 135, 344
Criticism with Clinical Notes. F. L. Griffith, M. D., . . . . . . . . . 67
Cures with a Single Dose. J. R. Haynes, M. D., . . . . . . . . . . 427

Dangers Arising from Public Funerals of those who have Died of Contagious Diseases. Circular No. 29, State Board of Health of Pennsylvania. Review of, . . . . . . . . 43
Daughter, The. By Wm. M. Capp, M. D., . . . . . . . . . . . . . . . . . 230
Davis, Frank S., M. D. Grafts, . . . . 38
Dean, D. H., M. D. Some Verifications of Similia, . . . . . . . . . . . . . 102
Dental Mirror, Notice of, . . . . . . . 90
Development of Nosode Practice in the Old School. Geo. H. Clark, M. D., 6
Dever, Dr. I. Gonorrhœa Again, . . . 173
Experience with Pneumonia, . . . 404
Diabetes. By Charles W. Purdy, M. D. Review of, . . . . . . . . . . . . 229
Diabetes, Lectures on. By Robert Saundby, M. D., . . . . . . . . . . . . . 135
Dietetic Gazette, The. Notice of, . . . 90
Dike, John, M. D. A Collection of Symptoms Going from Left to Right and from Right to Left, . . 107
Dios Chemical Co., The. Note, . . . . 341
Disposal of the Sewage of Public Edifices. Circular No. 20, State Board of Health Penna., The. Review of, . . . . . . . . . . . . . . . . . 43
Doctor, The. George H. Clark, M. D., 345
Dr. C. Carleton Smith's Nuggets. S. Lilienthal, M. D., . . . . . . . . . 66
Dudley, Pemberton, M. D. The American Institute of Homœopathy and the International Homœopathic Congress, . . . . . . . . . 121
Dunham, Geo. F., M.D. Clinical Verifications, . . . . . . . . . . . . . . 220
Dyspepsia, with Salty Taste. Dr. J. Kafka (S. L.), . . . . . . . . . . . 130
Dystocia, A New Procedure in. J. W. Thomson, M. D., . . . . . . . . . 246

Eczema, . . . . . . . . . . . . . . . . . 426

Editorials, 1, 47, 93, 139, 185, 233, 265, 297, 345, 377, 409, 441

Education in Homœopathy. Note, . . 46

Epilepsy. Proceedings of I. H. A., . . 203

Epithelioma. T. Dwight Stow, M. D., 211

Errors, Other, in Proceedings of I. H. A., . . . . . . . . . . . . . . . . . 232

Erysipelas, . . . . . . . . . . . . . . 425

Euphrasia, . . . . . . . . . . . . . . . 422

Eurich, Dr. C., Removal, . . . . . . . 408

Experience with Pneumonia. I. Dever, M. D., . . . . . . . . . . . . . . 404

Eye, Treatise on Diseases of. C. H. Angell, M. D. Review of, . . . 291

Eyermann, Dr., Removal, . . . . . . . 408

Fabiola, The, . . . . . . . . . . . . . 408

Farley, Robert, M. D. Gonorrhœa and Homœopathy, . . . . . . . . . . . 30

What are the Remedies? . . 393, 448

Fever. Its Pathology and Treatment. By Hobart Amory Hare, M. D. Review of, . . . . . . . . . . . . . 438

Fifth Annual Report of the State Board of Health and Vital Statistics of the Commonwealth of Pennsylvania. Review of, . . . . . . . 439

Fifth State Sanitary Convention, The. Note, . . . . . . . . . . . . . . . 135

Fincke, B., M. D. To Err is Human, to Forgive Divine, . . . . . . . 337

Translation of *The Organon*, . . . 477

Five Years' Experience in the New Cure of Consumption. By J. Compton Burnett, M. D. Review of, . . . . . . . . . . . . . . . . . 182

For Sale, . . . . . . . . . . . . . . . . 376

Foreign Objects, Method of Expelling from Digestive Tube, . . . . . . 226

Foster, L. P., M. D. Venereal Diseases, 219

PAGE

Fun for Doctors, . . . . . . . . . . 138, 184

Funerals, Public, Danger From, . . . 43

Future of Medicine. Geo. H. Clark, M. D., . . . . . . . . . . . . . . 8

Geraniaceæ, . . . . . . . . . . . . . . 168

Getting Married and Keeping Married. Review of, . . . . . . . . . . . 339

Gilbert, C. B., M. D.

Koch and His Discovery, . . . . . 202

Primary and Secondary Symptoms, and the Dose, . . . . . . . . . . 60

Syphilinum, . . . . . . . . . . . 24

Glen Mary Home, The. Note, . . . . . 264

Gonorrhœa Again. Dr. I. Dever, . . . 173

Gonorrhœa and Homœopathy. Robert Farley, M. D. . . . . . . . . . . 30

Gonorrhœa and Homœopathy. S. A. Kimball, M. D., . . . . . . . . . 31

Gonorrhœa and Homœopathy. J. C. White, M. D., . . . . . . . . . . 83

Gonorrhœa and Homœopathy. Defence of Dr. Allen. J. C. White, M. D., . . . . . . . . . . . . . 25

Gonorrhœa with Shotgun Treatment. C. M. Boger, M. D., . . . . . . . 400

Gonorrhœa and "Straight Homœopathy." Thos. Skinner, M. D., . 106

Government Indorsement of Professor Koch. Geo. H. Clark, M. D.. . . 190

Grafts. Frank S. Davis, . . . . . . . 38

Grand Rapids, Mich., College of Homœopathic Physicians and Surgeons, The. Note, . . . . . . . . . . . 376

Greater Diseases of the Liver, the. J. Compton Burnett, M. D., . . . . 476

Griffith, F. L., M. D. A Criticism, with Clinical Notes, . . . . . . . . 67

Guernsey, Wm. Jefferson, M. D. Medicinal Shock—Arnica, . . . . . . 248

Guiding Symptoms of our Materia Medica. Published by F. A. Davis. Review of, . . . . . . . 134

Hahnemann Hospital in Philadelphia. The New Building, . . . . . . . 137

Hahnemannian's Analysis Sheet. By M. A. A. Wolff, M. D. Review of, 92

Hahnemannian's Analysis Sheet, The. Notice of, . . . . . . . . . . . 184

Hahnemannian's Analysis Sheet, The. M. A. A. Wolff, . . . . . . . . . 227

Hahnemannian Association of Boston, 183

Hall, John, M. D.

Medical Persecution in British Columbia, . . . . . . . . . . . . 275

A Case of Skin Disease—Pediculus Corposis, . . . . . . . . . . . . 406

Hare, H. A., M. D. Fever, its Pathology and Treatment. Review of, 438

Hatfield, Walter S., M. D.

Lecture upon Homœopathy, . . . 52

Lecture upon the First Three Paragraphs of *The Organon*, . . . . . 150

Hawley, Dr. William. Obituary, . 261, 289

Haynes, J. R., M. D.

Cures with a Single Dose, . . . . . 427

Some Confirmed Symptoms, . . . . 214

Headache and Neuralgia, by J. L. Corning, M. D. Review of, . . . 42

Heart Failure. Geo. H. Clark, M. D., 267

Heath, Alfred, M. D.

British Medicinal Plants, 19, 79, 164, 200, 251, 368, 468

Dr. Heath's Plan for Studying Remedies, . . . . . . . . . . . . 178

PAGE

Hepar, . . . . . . . . . . . . . . . 429

Heredity, Health, and Personal Beauty. By John Shoemaker, M. D. Review of, . . . . . . . . 293

Hiccough. W. Steinrauf, M. D., . . . 24

"Higher Medical Education." Dr. Geo. H. Clark, . . . . . . . . . . 139

High Potencies. Provings and Clinical Observations with. M. Macfarlan, M. D., . . . . . . . . . . 449

Homœopathic Bibliography of the United States. By Thos. L. Bradford, M. D. Review of, . . . . . 263

Homœopathic Cures. S. L., . . . . . . 354

Homœopathic Dilutions. Walter M. James, M. D., . . . . . . . . . . 377

Homœopathic Medical College of Missouri, Commencement of, . . . . 231

Homœopathic Medical Council, The, 249, 330

Homœopathic Medical Society of Kansas. Note, . . . . . . . . . . . . 137

Homœopathic Medical Society of the State of Oregon. Note, . . . . . 344

Homœopathy and Allopathy, . . . . . 49

Homœopathy and Blood-Letting. By W. B. Clarke, M. D. Review of, . 43

Homœopathy Going to the Bow-Wows. The Eclectic Medical Journal, . 40

Homœopathy or Isopathy, Is It? Samuel Swan, M. D., . . . . . . 425, 443, 444

Homœopathy, Lecture Upon. Walter S. Hatfield, M. D., . . . . . . . . 52

Homœopathy Proven by Koch. . . . . 435

Homœopathy, The Teaching of in the Colleges. Edward F. Brady, M. D., . . . . . . . . . . . . . 39

Homœopathy, What It Is and What It Is Not. By Thomas Wildes, M. D. Review of, . . . . . . . . . . . . 339

Hospital, Children's Homœopathic, . . 184

How to Magnetize. James Victor Wilson. Review of, . . . . . . . . . 375

Hygiene, Text-Book of. Review of, . 340

Hypericaceæ, . . . . . . . . . . . . 166

Ice in the Sick Room. Note, . . . . . 232
Ice, Impure, . . . . . . . . . . . . . 343
Illinois State Society, . . . . . . . . . 480
International Hahnemannian Association Meeting, The. George H. Clark, M. D., . . . . . . . . . . 297
An Open Personal Letter to the Members of the. James B. Bell, 434
Proceedings of, Epilepsy, . . . . . 203
Surgical Operations upon the Ovaries, . . . . . . . . . . . . . . 143
Il. Secolo Omiopatico. Notice of, . . . 407
Impure Ice, . . . . . . . . . . . . . 343
Indiana Institute of Homœopathy, The, 183, 296
Indigestion in Infants. Nathan Cash, M. D., . . . . . . . . . . . . . . 394
Infant Feeding. W. A. D. Pierce, M.D., 437
Infant Food. Geo. H. Clark, M. D., . 237
Infant Food, . . . . . . . . . . . . . 335
Infants, Indigestion in. Nathan Cash, M. D., . . . . . . . . . . . . . 394
In Memoriam. S. Lilienthal, M. D., . 473
In Memoriam. P. P. Wells, M. D., . . 444
Insane Asylum, Private, . . . . . . . . 478
Instructions in the True Principles of Homœopathy. E. J. Lee, M. D., 49
International Congress. Final Notice, 255
International Homœopathic Congress, 137, 229, 306

PAGE
International Congress and the Institute, The. W. M. James, M. D., 229
International Hahnemannian Association, Annual Meeting of 1891, 136, 184, 232, 411
Other Errors in Proceedings of . . 232
International Medical Annual. Announcement of, . . . . . . . . . 135
International Medical Annual and Practitioners' Index, The. Review of, . . . . . . . . . . . . . . 229
Is it Homœopathy or Isopathy? 425, 443, 444
Isopathy and other Patholgical Prescribing. E. J. Lee, M. D., . . . 192

Jackson, J. H., M. D. An Instructive Lesson, . . . . . . . . . . . . . 21
Jahr, G. H. G., M. D., Clinical Guide, . 474
Jamacia Ginger, . . . . . . . . . . . . 403
James, Dr. Bushrod W,, . . . . . . . . 478
James, Walter M., M. D.
Dr. Wells on Intermittent Fever. 237
Dr. Wells on Intermittent Fever. Apology. . . . . . . . . . . . . . 229
Homœopathic Dilutions, . . . . . 377
The International Congress and the Institute, . . . . . . . . . . 229
Is it Homœopathy or Isopathy? . 443
Salutatory Editorial, . . . . . . . 1
Pathological Provings, . . . . . . 409
Journal of Balneology and Dietary, The. Review of, . . . . . . . . 295

Kafka. Dr. J. Dyspepsia with Salty Taste, . . . . . . . . . . . . . . . 130
Kali-carb., . . . . . . . . . . . . . . . 419
Kali-iod., . . . . . . . . . . . . . . 217
Kansas City Hospital, The. M. A. A. Wolff, . . . . . . . . . . . . . . . 435
Kansas City Medical Society, . . . . . 478
Kennedy, A. L., M. D. Symptoms Removed by Remedies During Treatment of Cases, . . . . . . . 419
Kimball, S. A., M. D. Gonorrhœa and Homœopathy, . . . . . . . . . . 31
King's Journal Directory. Note, . . . 137
Klip, Binding. Ballard's . . . . . . . 477
Koch's Discovery. Geo. H. Clark, M. D., . . . . . . . . . . . . . . 5
Koch and his Discovery. C. B. Gilbert, M. D., . . . . . . . . . . . . . . . 202
Koch. Homœopathy proven by, . . . . 435
Koch's Lymph and Swan's Tuberculinum. Geo. H. Clark, M. D., . . . 185
Koch's Lymph and Swan's Tuberculinum. E. J. Lee, M. D., . . . . . 238
Kraft, Dr. Frank. Note, . . . . . . . 408
Removal, . . . . . . . . . . . . . 264
Krusen, E. A., Dr. The Homœopathic Medical Council, . . . . . . . . . 330

Labor. See Confinement.
Lac Caninum. A Study of. D. C. Perkins, M. D., . . . . . . . . . . . 84
Lacto Cereal Food, . . . . . . . . . . 477
La Grippe Again. Wm. Steinrauf, M. D., . . . . . . . . . . . . . . . 122
Lee, Dr. Benjamin. Note, . . . . . . . 135
Lee, E. J., M. D.
In Memoriam. P. P. Wells, M. D., . . . . . . . . . . . . . . . 444
Instructions in the True Principles of Homœopathy, . . . . . . . . 49
Isopathy and other Pathological Prescribing, . . . . . . . . . . . 192
Lee, E. J., M. D.
Kock's Lymph and Swan's Tuberculinum, . . . . . . . . . . . . 238
How to use a Repertory, . . . . . . 9
Left to Right and Right to Left. A Collection of Symptoms going from. John Dike, M. D., . . . . 107
Leguminosæ, . . . . . . . . . . . 201, 251
Lesson, An Instructive. J. H. Jackson, M. D., . . . . . . . . . . . . 21
Letter, Open Personal, to Members of I. H. A. J. B. Bell, M. D., . . . . 434
Lilienthal, Samuel, M. D.
Carduus Marinus. Dr. Kunze, . . 221
Dr. C. Carleton Smith's Nuggets, . 66
Cases from Practice. Dr. Lorbacher, . . . . . . . . . . . . . 224
Chloroform, Treatment in Typhoid Fever. Dr. Hepp, . . . . . . . 181
Chronic Intoxication from Essences, as Wermuth, etc., . . . . 466
Homœopathic Cures, . . . . . . . 354
In Memoriam, . . . . . . . . 417, 473
A Lycopodium Cure. Dr. H. Goullon, . . . . . . . . . . . . . 244
Ophthalmia Neonatorum and its Treatment, . . . . . . . . . . . 402
Remedies for Alternating Disease, 284
Rheumatism of Kalmia, . . . . . 176
Some Queer Symptoms of Lyssa, . 245
London, B., M. D. On Powers of Absorption of Urinary Bladder, . . 474
Long Island College Hospital, . . . . . 480
Lutze. F. H., M. D. Six Clinical Cases, . . . . . . . . . . . . . . 256
Lycopdium Cure. A. Dr. H. Goullon. S. L., . . . . . . . . . . . . . . 244
Lyssa. Some Queer Symptoms of. Dr. Proell. S. L., . . . . . . . . . . . 245

Macfarlan, Malcolm, M. D. Provings and Clinical Observations with High Potencies, . . . . . . . . . 449
Mag-phos., . . . . . . . . . . . . . . 421
Malvaceæ, . . . . . . . . . . . . . . . 165
Materia Medica Study. W. A. Yingling, M. D., . . . . . . . . . . . 387
Materia Medica and Therapeutics, John V. Shoemaker, M. D. Review of, . . . . . . . . . . . . . 263
Mattison Prize, The. Note, . . . . . . 343
Medical Argus, The. Notice of, . . . . 44
Medical Bulletin Visiting List, or Physicians' Call Record, The. Notice of, . . . . . . . . . . . . . . 44
Medical Legislation. C. H. Oakes, M. D., . . . . . . . . . . . . . . . . 349
Medical Persecution in British Columbia. John Hall, M. D., . . . . . 275
Medical Symbolism. By Thos. S. Sozonskey, M. D. Review of, . . . 291
Medicine, The Future of. George H. Clark, M. D., . . . . . . . . . . . 8
Mental Suggestion. By Dr. J. Ochorowicz. Review of, . . . . . . . . 439
Mercurius-iod., . . . . . . . . . . . . 216
Method of Expelling Foreign Objects Taken into the Digestive Tube, A, . . . . . . . . . . . . . . . 226
Michigan State Homœopathic Medical Society, The. Note, . . . . . . . 376
Minnesota State Homœopathic Institute. Note, . . . . . . . . . . . 296
Miscarriage. Geo. H. Clark, M. D., . . 265
Missouri Institute of Homœopathy, 136, 478
Munson, Dr., . . . . . . . . . . . . . 341

PAGE
National Conservatory of Music of America, The. Notice of, . . . . 342
Natr-mur., . . . . . . . . . . . . . 220, 422
Nervous System, Twelve Lectures upon. By Ludwig Edinger. Review of, 294
Neuralgia, A Severe Case of. Dr. A. B. Syontagh, . . . . . . . . . . 76
Never, "The Disease per se." A. H. Tompkins, M. D., . . . . . . . . . 177
New Food, A. Note, . . . . . . . . . 341
New York Homœopathic Union, The, 277, 366
New York Medical College and Hospital for Women. Note, . . . . . 341
Noe, A. T., M. D.
Advice Wanted, . . . . . . . . . . . 254
Dr. Noe's Case in June Number, . 371
Noe's Case in June Number, Dr., A. T. Noe, M. D., . . . . . . . . . . . 371
Wm. Steinrauf, M. D., . . . . . . . 288
North American Practitioner. The, Review of, . . . . . . . . . . . . 295
Norton, Dr. A. B. Note, . . . . . . . 184
Nosode Practice in the Old School, The Development of. Geo. H. Clark, M. D., . . . . . . . . . . . . . . . 6
Notes and Notices, 46, 134, 183, 231, 264, 296, 341, 376, 408, 476
Notes from Past Meetings of the Homœopathic Medical Council, . . . 249
Nuggets. C. Carleton Smith, M. D., 16, 196
Dr. C. Carlton Smith's S. Lilienthal, M. D., . . . . . . . . . . . . . . . 66
An Answer to Dr. Lilienthal. C. Carleton Smith, M. D., . . . . . 175
Nux-vom., . . . . . . . . . . . . . . . 221
In Labor. W. A. Yingling, M. D., 72

Oakes, C. H., M. D. Medical Legislation, . . . . . . . . . . . . . . . 349
Obituary. Dr. Wm. A. Hawley, . . . . 261
Dr. Samuel Lilienthal, . . . . . . 417
Dr. Alfred Isaac Sawyer, . . . . . 262
Dr. David Smith, . . . . . . . . . 262
Ochorwicz, Dr. J., Mental Suggestion, . 439
Ohio State Society, . . . . . . . . . . 480
Open Court Publishing Company, The. Note, . . . . . . . . . . . . . . . 46
Ophthalmia Neonatorum. Geo. H. Clark, M. D., . . . . . . . . . . . 266
And its Treatment. S. Lilienthal, 402
Orificial Surgery, . . . . . . . . . . . 478
*Organon*, Lecture upon the first Three Paragraphs of the. Walter S. Hatfield, M. D., . . . . . . . . . . 150
Other Errors. Note. . . . . . . . . . 232
Our Animal Friends, . . . . . . . . . 474
Ovaries, Surgical Operations upon the. Trans. of I. H. A., . . . . . . . . 143
Oxalidaceæ. . . . . . . . . . . . . . 200
Oxygen and Hydrogen. A Correction. E. V. Ross, M. D., . . . . . . . . . 437

Pathological Provings. Walter M. James, M. D., . . . . . . . . . . . 409
Pediculis Corposis. A Case of Skin Disease. John Hall, M. D., . . . 406
Penna. State Board of Health. Dangers from Public Funerals, . . . 43
Fifth Annual Report, . . . . . . . 439
Perkins, D. C., M. D. A Study of Lac-caninum, . . . . . . . . . . . . . 84
Physician's All-Requisite Time and Labor-Saving Account Book. Review of, . . . . . . . . . . . . . . 45
Pierce, W. A. D., M. D. Infant Feeding, 437

PAGE
Pneumonia, Experience with. I. Dever, M. D., . . . . . . . . . . . . . 404
Pocket Anatomist, The. By C. Henri Leonard, M. D. Review of, . . . 372
Pocket Materia Medica and Therapeutics, The. By C. Henri Leonard, M. D. Review of. . . . . . . . . 294
Poisoning by Rhus-toxicodendron. W. A. Yingling, M. D., . . . . . . . . 433
Poisoning by Rhus-tox., . . . . . . . . 334
Pomeroy, T. F., M. D. Science and Old Medicine Contrasted, . . . . 457
Post Graduate Course, The, . . . . . . 135
Post Graduate Clinical Charts, The. By Drs. Bailey and Linsley. Review of, . . . . . . . . . . . . . . 263
Post-Mortems. What to Look for and How to make them. By A. H. Newth, M. D. Review of, . . . 45
Practical Manual of Gynecology. By G. R. Southwick, M. D. Review of, . . . . . . . . . . . . . . 374
Preston, Mahlon, M. D. Dr. Preston's Case of Syphilis, . . . . . . . . . 36
Primary and Secondary Symptoms and The Dose. Chas. B. Gilbert, M. D., . . . . . . . . . . . . . . . 60
Principles of Surgery. By N. Senn, M. D. Note of, . . . . . . . . . 407
Printer's Ink, . . . . . . . . . 341, 408, 477
Provings and Clinical Observations with High Potencies. Malcolm Macfarlan, M. D., . . . . . . . . . 449
Psora Theory and Dr. Reuter's Observations, The. Geo. H. Clark, M. D., . . . . . . . . . . . . . . . 237
Dr. Geo. H. Clark, . . . . . . . . . . 93
Psorinum, . . . . . . . . . . . . . . . 422
Purdy, Chas. W., M. D. Diabetes, . . 229

Reason Why, The. Samuel Swan, M. D., . . . . . . . . . . . . . . . 161
Recognition of Women in the Profession, The. T. Dwight Stow, M. D., . . . . . . . . . . . . . . . 179
Reed & Carnrick, Messrs. Note, . . . 408
Remedies for Alternating Diseases. S. L., . . . . . . . . . . . . . . . . 284
Remedies, What are the. Robert Farley, M. D., . . . . . . . . . . . . . 393
Remarkably Successful Operation, A, 343
Removals, . . . . . . . . . . . . 264, 476
Renal Colic—Berberis-vulg. S. W. Cohen, M. D., . . . . . . . . . . 431
Repertories, Private. W. A. Yingling, M. D., . . . . . . . . . . . . . . 124
Repertory, How to use A. Ed. J. Lee, M. D., . . . . . . . . . . . . . . . 9
Report of the Bureau of Organization, Registration, and Statistics of the American Institute of Homœopathy. By Thomas Franklin Smith, M. D. Review of, . . . . 44
Report of Committee on Vital Statistics, State Board of Health of Penna. Review of, . . . . . . . . . . . . 43
Resection of the Optic Nerve. By L. Webster Fox, M. D., . . . . . . . 293
Retrospect, A., . . . . . . . . . . . . 441
Rheumatism of Kalmia, The. S. Lilienthal, M. D., . . . . . . . . . . 176
Rhinoplasty. By Trebhovandas Motichand, M. D. Review of, . . . . 133
Rhode Island Hom. Society, The, . . . 264
Rhus-tox., . . . . . . . . . . . . . . . 214
Rhus-tox. Poisoning, . . . . . . . . . 334

PAGE

Rhus-tox., Poisoning by. W. A. Yingling, M. D., . . . . . . . . . . . . 433
Right to Left, and Left to Right, Symptoms Going From. John Dike, M. D., . . . . . . . . . . . . . . . 107
Rogers, Dr. Note, . . . . . . . . . . . 344
Rosaceæ, . . . . . . . . . . . . . . . 251
Ross, E. V., M. D. Oxygen and Hydrogen—A Correction, . . . . . . . 437

Salutatory Editorial. Geo. H. Clark, M. D., . . . . . . . . . . . . . . . 4
 Walter M. James, M. D., . . . . . 1
Sanguinaria, . . . . . . . . . . . . . 422
Sanitary Convention, Fifth State, . . . 135
Saundby, Robert, M. D. Lectures on Diabetes, . . . . . . . . . . . . 135
Sanitary Era, The. Notice of, . . . 91, 339
Sawyer, Dr. Alfred. Obituary, . . . . 262
Science and Old Medicine Contrasted. T. F. Pomeroy, M. D., . . . . . . 457
Scientific Medicine in its Relation to Homœopathy. Prof. Bakody, . . 475
Senn's Principles of Surgery. Review of, . . . . . . . . . . . . . . 407
Sepia, . . . . . . . . . . . . . . . . . 423
Sexual Health. By Henry G. Hanchett, M. D. Review of, . . . . . . 438
Sexual Neurasthenia. By Geo. M. Beard, M. D. Review of, . . 135, 374
Sherbino. Geo. W., M. D. Clinical Verifications, . . . . . . . . . . 69
Shock, Medicinal. W. J. Guernsey, M. D., . . . . . . . . . . . . . . . 248
Shoemaker, John V., M. D. Heredity, Health, and Personal Beauty. Review of. . . . . . . . . . . . . . 293
 Materia Medica and Therapeutics. Review of, . . . . . . . . . . . . . 263
Single Dose, Cases with a. J. R. Haynes, M. D., . . . . . . . . . . . . . . . 427
Six Clinical Cases. F. H. Lutze, M. D., . . . . . . . . . . . . . . . . 256
Skin Disease, a Case of. John Hall, M. D., . . . . . . . . . . . . . . . . 406
Skinner, Dr. Note, . . . . . . . . . 343, 408
Skinner, Thos., M. D. Gonorrhœa and "Straight Homœopathy," . . . 106
Smith, C. Carleton, M. D. Nuggets, 16, 196
 Nuggets. An Answer to Dr. Lilienthal, . . . . . . . . . . . . . 175
Sozinskey, Thomas, M. D. Medical Symbolism, . . . . . . . . . . 291
Smith, Dr. David. Obituary, . . . . . 262
Standard Dictionary of the English Language, The. Notice of, . . . 407
Stannum, . . . . . . . . . . . . . . . . 424
Stanton, L. M., M. D. The New York Homœopathic Union, . . . . 277, 366
State Board of Health Bulletin of Tennessee for Nov. Review of, . . . 42
Staunton, Virginia. By Armistead C. Gordon, Notice of, . . . . . . . 134
Steinrauf, W., M. D.
 Cholera Infantum, . . . . . . . . 470
 Hiccough, . . . . . . . . . . . . . 24
 La Grippe Again, . . . . . . . . . 122
 Dr. Noe's Case in June No., . . . . 288
Stillman & Hosmer, Drs. Note, . . . . 341
Stilson, W. C., M. D. An Accidental Proving of Balm of Gilead Buds, 88
Stow, T. Dwight, M. D.,
 A Brief Retrospect of Matters Surgical, . . . . . . . . . . . . . . 206
 A Few Cases in Surgical Practice, . . . . . . . . . . . . . . . . 210

PAGE

Stow, T. Dwight, M. D.
 Epithelioma, . . . . . . . . . . . 211
 A Queer Case, . . . . . . . . . . . 212
 The Recognition of Women in the Profession, . . . . . . . . . . . 179
 Veneral Diseases. . . . . . . . . . 209
Sulphur, . . . . . . . . . . . . . . . . 221
Superstitions. Geo. H. Clark, M. D., . 233
Surgical Matters. A Brief Retrospect of, . . . . . . . . . . . . . . . . . 206
Surgical Practice. A Few Cases in, . . 210
Swan's Theory. Dr. Geo. H. Clark, M. D., . . . . . . . . . . . . . . . . . 5
Swan, Samuel., M. D.
 Curative Treatment of Bacterial Diseases (so-called), . . . . . . . 241
 Is it Homœopathy or Isopathy ? . 425
 The Reason Why, . . . . . . . . . 161
Symbolism, Medical. See Medical Symbolism.
Symptoms going from Left to Right, and from Right to Left. A Collection of. John Dike, M. D., . . 107
Symptoms. Primary and Secondary. M. W. Vandenburg, M. D., . . . 116
Symptoms Removed by Remedies During Treatment of Cases. A. L. Kennedy, M. D., . . . . . . . . . 419
Symptoms. Some Confirmed. J. R. Haynes, M. D., . . . . . . . . . . 214
Syontagh. A. V., M. D. A Severe Case of Neuralgia, . . . . . . . . . . . 76
Syphilinum. Chas. B. Gilbert, M. D., 24
Syphilinum. Thomas Wildes. M. D., 267, 359
Syphilis. Dr. Preston's Case of. Mahlon Preston, M. D., . . . . . . 36

Taber, Dr. Geo. A., . . . . . . . . . . 477
Texas State Society, . . . . . . . . . . 478
Text Book of Hygiene. By George H. Rohé, M. D. Review of, . . . . 340
Thomson, J. W., M. D. A New Procedure in Dystocia, . . . . . . . . 246
Thomson, J. W., M. D. The Transactions of the I. H. A., . . . . . 132
Thompson, Dr. Landreth W. Note, . . 136
Three Thousand Questions on Medical Subjects. Review of, . . . . . . . 440
To Err is Human, to Forgive Divine. B. Fincke, M. D., . . . . . . . . . . 337
Tompkins, A. H., M. D. Never "The Disease per se," . . . . . . . . . 177
Transactions of California State Society. Review of, . . . . . . . . . 43
Transactions of the I. H. A., The. J. W. Thomson, M. D., . . . . . . . . 132
Transactions of the Homœopathic Medical Society of the State of New York, 1890. By John L. Moffat, M. D. Review of, . . . . . . 373
Transactions of the Homœopathic Medical Society of Penna. Review of, . . . . . . . . . . . . 438
Treatise on Diseases of the Eye. By Henry C. Angell, M. D. Review of, . . . . . . . . . . . . . . . . . 291
Treatise on Headache and Neuralgia. By I. Leonard Corning, M. D. Review of, . . . . . . . . . . . . . . 42
Treatise on Practical Anatomy. H. C. Bœnning, M. D., . . . . . . . . . 476
Twelve Lectures on the Structure of the Central Nervous System. By Dr. Ludwig Edinger. Review of, . . . . . . . . . . . . . 294

PAGE
Urinary Bladder. On Powers of Absorption of. B. London, M. D., . 474

Vacation Time, with Hints on Summer Living. By H. S. Drayton, M. D. Review of, . . . . . . . . . 372
Vandenburg, M. W., M. D. Primary and Secondary Symptoms, . . . 116
Venereal Diseases. L. P. Foster, M. D., . . . . . . . . . . . . . . . . . 219
T. Dwight Stow, M. D., . . . . . . 209
Verat-alb., . . . . . . . . . . . . . 421, 424
Verifications of Similia, Some. D. H. Dean, M. D., . . . . . . . . . . . . 102
Victim of Addison's Disease, A. Note, 342

Waggoner, Dr. G. J. Removal, . . . . 408
Wells, P. P., M. D., In Memoriam, . . 444
Wells on Intermittent Fever, Dr. W. M. James, M. D., . . . . . . . . . . . 237
Apology. W. M. James, M. D., . . 299
Wells, Dr. L. B. Removal, . . . . . . 408

PAGE
What are the Remedies? Robert Farley, M. D., . . . . . . . . . . . . . 393, 448
White, J. C., M. D. Gonorrhœa and Homœopathy, . . . . . . . . . 25, 83
Wildes, Thos., M. D.
Homœopathy, What it is and What it is Not. Review of, . . . . . . 339
Syphilinum, . . . . . . . . . . . 267, 359
Wolff, M. A. A., M. D.
Death of, . . . . . . . . . . . . . . 476
The Hahnemannian's Analysis Sheet, . . . . . . . . . . . 92, 184, 227
The Kansas City Hospital, . . . . 435
Woman's Homœopathic Hospital. Notice of, . . . . . . . . . . . . . . . 46
Women, A Discouraging Opinion of, . 180
Advice to. Review of, . . . . . . . 91

Yingling, W. A., M. D.

Materia Medica Study, . . . . . . . 387

Nux-vomica in Labor, . . . . . . 72

Poisoning by Rhus-toxicodendron, 433

Private Repertories, . . . . . . . . 124

VOL. XI. JANUARY, 1891. No. 1.

THE

### HOMŒOPATHIC PHYSICIAN,

### A Monthly Journal of Homœopathic Materia Medica and Clinical Medicine.

"*He is a freeman whom truth makes free, and all are slaves beside.*"
"*Seek the Truth: come whence it may, cost what it will.*"

EDITED AND PUBLISHED BY

WALTER M. JAMES, M. D.,

AND

GEORGE H. CLARK, M. D.

SURGEON GENL'S OFFICE, LIBRARY.

NOTICE.

*All articles for publication, books for review, busi[illegible]cations, etc., must be sent to Editors, 1125 Spruce Street, Philadelphia.*

*Subscribers will please notice that this Journal will be sent until arrears are paid and it is ordered discontinued.*

PHILADELPHIA:

No. 1125 SPRUCE STREET.

